# Vaccine Hesitancy: Contemporary Issues and Historical Background

**DOI:** 10.3390/vaccines10101595

**Published:** 2022-09-22

**Authors:** Rina Fajri Nuwarda, Iqbal Ramzan, Lynn Weekes, Veysel Kayser

**Affiliations:** Sydney Pharmacy School, Faculty of Medicine and Health, The University of Sydney, Sydney, NSW 2006, Australia

**Keywords:** vaccines, vaccine hesitancy, anti-vaccine movement, vaccination

## Abstract

Vaccination, despite being recognized as one of the most effective primary public health measures, is viewed as unsafe and unnecessary by an increasing number of individuals. Anxiety about vaccines and vaccination programs leading to vaccine hesitancy results from a complex mix of social and political influences, cultural and religious beliefs, the availability of and ability to interpret health and scientific information, and personal and population experiences of health systems and government policies. Vaccine hesitancy is becoming a serious threat to vaccination programs, and was identified as one of the World Health Organization’s top ten global health threats in 2019. The negative impact of anti-vaccination movements is frequently cited as one of the major reasons for rising vaccine hesitancy amongst the general public world-wide. This review discusses the various issues surrounding vaccine hesitancy and the anti-vaccine movement, starting with the definitions of vaccine hesitancy and the anti-vaccine movement in their early history and in the modern era, before discussing the key drivers of vaccine hesitancy, particularly across different regions of the world, with a focus on various countries with low-, middle-, or high-income economies with different socio-economic populations. The review concludes with the impact of vaccine hesitancy on herd immunity and social, psychological, and public health measures to counter vaccine hesitancy.

## 1. Definition and Theoretical Framework

Vaccination is one of the most significant public health achievements, having contributed to the eradication of smallpox and the control of many infectious diseases such as rubella, diphtheria, and polio globally. For many diseases, the success of this strategy is dependent on public acceptance of vaccination. While vaccination has been shown to be very effective in reducing the global burden of infectious diseases and deaths associated with vaccine-preventable diseases, concerns that undermine public trust and the acceptance of vaccines continue. If such confidence continues, there is a risk of the resurgence of vaccine-preventable diseases because of delays, rejections, and disruptions to vaccine development, delivery, availability, and further research [1,2,3].

**Figure 1:**
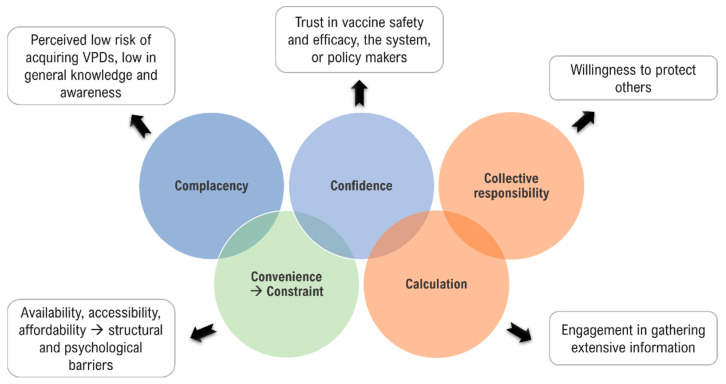
Vaccine hesitancy model: the 3Cs to 5Cs. In 2014, the WHO’s SAGE group determined factors that could influence vaccine hesitancy as the 3Cs: complacency, confidence, and convenience. The 3Cs model was revised in 2018 and became the 5Cs model, replacing convenience with constraint and adding calculation and collective responsibility (figure is adapted from text in reference [9]).

The prevalence of vaccine hesitancy over several decades has led to it being listed as one of the top 10 global health threats by the World Health Organization (WHO) in 2019 [3,4]. In 2014, the WHO’s Strategic Advisory Group of Experts on Immunization (SAGE) defined vaccine hesitancy as: *“Vaccine hesitancy refers to delay in acceptance or refusal of vaccines despite availability of vaccination services. Vaccine hesitancy is complex and context-specific, varying across time, place, and vaccines. It is influenced by factors such as complacency, convenience, and confidence”* [5,6]. During the last decade, an important amount of research has been conducted regarding vaccine hesitance and the multiple factors influencing an individual’s decision to accept or not accept a vaccine. In this regard, the WHO Strategic Advisory Group of Experts on Immunization (SAGE) proposed three categories to study these factors: complacency (not viewing diseases as high-risk and vaccination as important), convenience (practical hurdles), and confidence (a lack of trust in vaccine safety and effectiveness), referred to as the 3Cs model. The concept of vaccine hesitancy does not apply in situations where vaccine uptake is low due to the unavailability of vaccines, lack of access to vaccines, unacceptable travel/distance to immunization clinics, and poor communication [5]. In 2016, a broader nomenclature spanning the dimensions of access, affordability, awareness, acceptance, and activation was suggested for the vaccine uptake determining factors, known as the 5As [7]. Based on both empirical and theoretical research, the 3Cs model was revised in 2018, emphasizing the importance of more than just the concept of confidence, and emerged as the 5Cs model: confidence, complacency, constraints (adjustment of the term convenience to now include both structural and psychological barriers), calculation (preference for deliberation), and collective responsibility (communal orientation) [8]. Figure 1 shows the adjustment of the vaccine hesitancy model from 3Cs to 5Cs.

While the SAGE concept was recognized, the reasons for vaccine hesitancy were more complex and varied, with some focused on community issues and others on the systems and health practitioners. A lack of validated surveys assessing hesitation in low- and middle-income countries was another key finding. Therefore, the WHO, UNICEF, and partners formed a working group focused on behavioral and social drivers of vaccination. They developed and validated survey techniques to assess vaccine hesitancy in high-, middle-, and low-income countries. This working group created a behavioral and social driver framework with four components: thinking and feeling (perceived disease risk and vaccine confidence), social processes (social norms and provider recommendation), motivation (intention to obtain recommended vaccines), and practical issues (availability, affordability, ease of access, service quality, and respect from provider). The Royal Society of Canada (RSC) Task Force on COVID-19 sub-group on vaccine acceptance established a more comprehensive model that emphasized the complexity, interconnectedness, and diverse range of factor categories. This adapted framework focused on four primary domains that drove vaccine uptake, including trust in vaccines (people and communities, healthcare workers (HCWs), accurate and trustworthy vaccination knowledge, and the healthcare system and public health programs) [9].

As the WHO has recognized, vaccine hesitancy is one of the most serious threats to global health. Vaccine hesitancy can lead to the failure of either establishing or maintaining the longevity of herd immunity, which is the requirement for the vaccination of a large proportion of the total population. The main concern with vaccine hesitancy and insufficient herd immunity is that unvaccinated individuals can act as virus reservoirs. They can cause more outbreaks, delaying efforts to control the spread of a virus to the entire population [10]. Upon achieving herd immunity through safe and efficient vaccines, diseases become less prevalent. Additionally, achieving herd immunity also aims to keep vulnerable individuals who cannot be vaccinated (e.g., due to health issues such as vaccine allergies) safe and protected against diseases. Individuals can also be left vulnerable to infections (such as tetanus or shingles), for which no herd immunity exists [11]. The percentage of individuals who need to be immunized against diseases to achieve herd immunity varies with the disease; herd immunity for measles requires the vaccination of ~95% of a population, while, for polio, the threshold is ~80% [12].

## 2. Early Vaccine Hesitancy

Resistance to vaccination is not a recent occurrence. Since the first smallpox vaccine was developed by Edward Jenner in 1796, skepticism and suspicions about vaccines and the motivations behind their use have existed [13].

While Jenner is recognized as contributing to immunization and the eventual eradication of smallpox, variolation was practiced prior to his discovery. However, variolization during early times was neither safe nor consistent; poorly trained practitioners could misdiagnose a donor’s chickenpox lesions as smallpox lesions and there were concerns that recipients would catch disseminated smallpox, infect others or contract diseases such as syphilis [14].

Inoculation was initiated in America during the smallpox epidemic, which hit Boston and other parts of Massachusetts in 1721 [14]. Fatality rates of 2% in variolated versus 14.9% for those infected naturally were reported [15,16]. However, English doctors questioned the extent and longevity of this effect [14].

Edward Jenner advocated for using the milder cowpox virus in a child to stimulate an immune response in 1796 after observing that milkmaids rarely contracted smallpox. Jenner tested his hypothesis on his gardener’s son [17] and, subsequently, on 23 more individuals [18]. In 1797, he presented his experiment to the Royal Society, who rejected it, citing insufficient evidence and the revolutionary nature of his results.

Jenner’s eventual publication of the results drew immediate public criticism and significant opposition. The local clergy argued that mixing animal matter with human flesh was a direct violation of God’s will. Others expressed concerns that the vaccine would cause “Cow-Mania”, using illustrations of an ox-faced boy and an elderly lady who allegedly grew horns after being vaccinated [19].

The 1853, the British Compulsory Vaccination Act announced the smallpox vaccine to be mandatory for infants during their first 3 months of life, creating the world’s first mandatory vaccination program and sparking widespread resistance; riots broke out in several UK towns [20]. The 1867, the act prescribed the mandatory vaccination age to 14 years, with penalties for non-compliance. Opponents of the 1867 law cited concerns about personal freedom and choice, and, in response, the Anti-Compulsory Vaccination League was founded the same year in London.

Numerous anti-vaccination publications emerged in the 1870s and 1880s, and anti-vaccination movements started to appear across Europe. By 1872, vaccination rates in Stockholm had fallen to ~40%, compared to ~90% in the rest of Sweden. However, a major smallpox epidemic occurred in 1874 and a greater uptake of vaccination avoided a further epidemic [21,22].

Anti-vaccination sentiments also grew in the United States (US) towards the end of the 19th century. Anti-vaccination movements arose as states attempted to enforce or pass new vaccination laws. Anti-vaccination societies sprung up around the USA and activists succeeded in repealing mandatory vaccination laws in various states [21,22].

When smallpox hit Cambridge, Massachusetts, in 1902, the city’s health board mandated all adults to be vaccinated. In 1905, during *Jacobson v Massachusetts*, where a citizen challenged the state’s authority to restrict personal freedom for public health reasons, the US Supreme Court ruled that the state may be justified in restricting individual liberty under the pressure of great dangers to ensure public safety [23].

## 3. Vaccine Hesitancy and Anti-Vaccine Movement(s) in the Modern Era

Between 1920 and 1970, new vaccines for tuberculosis, yellow fever, whooping cough, tetanus, and polio were introduced, dramatically lowering childhood mortality. Polio, which either paralyzed and/or killed children, was a major public health focus in the 1950s. Significant declines in polio following the introduction of the Salk vaccine in 1954 bolstered public acceptance of vaccines in general [24,25]. However, since the beginning of vaccines being marketed, public concerns about their efficacy and overall safety have existed.

### 3.1. Polio Vaccine: The Cutter Incident and Simian Virus 40 (SV40)

Following the introduction of the polio vaccine, one of the worst catastrophes occurred in the US. In 1955, despite successful mandatory safety testing, several batches distributed to the public contained the active polio virus. Over 250 cases of polio were linked to Salk’s vaccine manufactured by a small, family-owned company (Cutter Laboratories) [26]. Several vaccine lots were released that did not contain a fully inactivated polio virus, but instead included a live, active polio virus. One hundred and twenty thousand children received this vaccine with active polio viruses; seventy thousand children contracted mild polio from the vaccine, two hundred became permanently disabled, and ten died. This Cutter incident provided the groundwork for distrust in the pharmaceutical industry [27]. While the vaccine was recalled, this incident changed the way vaccines were manufactured and regulated [26].

Another concern surfaced between 1955 and 1963, when it was estimated that 10–30% of polio vaccines in the US were contaminated with SV40, suspected to cause human cancers. This virus was present in monkey kidney cell cultures, used to produce polio vaccines at the time. Following this contamination, the testing of all new polio vaccine lots to ensure that they were free of SV40 was mandated. No vaccines are used currently that include the SV40 virus, although a direct link between contaminated polio vaccines and cancer was never demonstrated [26].

### 3.2. Swine Flu Vaccine and Guillain-Barré Syndrome (GBS)

In 1976, in response to a potential influenza pandemic, a campaign to encourage vaccination against swine flu was launched. Over 45 million individuals received the vaccine, and there was a slight increase in GBS cases (one additional GBS case per 100,000 individuals vaccinated) [28].

Subsequent research yielded inconsistent results: from no proven link [29,30,31] to a risk of one additional GBS case per million vaccinated persons [32,33]. This information is publicly available and included in the recommendations of the Centers for Disease Control and Prevention (CDC) for influenza vaccines [34]. An elevated risk of GBS following influenza infection, several times more than the risk following influenza vaccination, has been reported [35]. GBS complications from vaccinations are extremely rare, highlighting that education programs are required to assist individuals to appropriately assess the benefits of vaccination versus risks of potential harms [36].

### 3.3. The Diphtheria, Tetanus, and Pertussis (DTP) Vaccine Controversy

In the mid-1970s, there was controversy over the safety of the diphtheria, tetanus, and pertussis (DTP) vaccination program. After being used for more than 20 years, in 1974, a retrospective study was published which described 36 children who suffered severe neurological complications with DTP [37]. Tragic stories of profoundly retarded children allegedly harmed by the vaccine were dramatized in the media, and concerned parents formed the Association of Parents of Vaccine-Damaged Children (APVDC). The independent Joint Commission on Vaccination and Immunization (JCVI) launched the National Childhood Encephalopathy Study (NCES) to determine whether vaccination was associated with an increased risk of encephalopathy. The JCVI concluded that the risk was extremely low, but negative public sentiment and uncertainty within the medical profession led to a rapid decline in the immunization rate; shortly after, the first of three major epidemics of whooping cough broke out in the UK [38].

A second controversy with DTP vaccines arose in the US in 1982, and public opinion began to shift following an NBC documentary titled *DPT: Vaccine Roulette* and a book called *A Shot in the Dark* [39]. The documentary alleged that children exhibited seizures and permanent brain damage from the vaccine [25]. Seizures were a rare adverse reaction to the vaccine, but no long-term effects had been demonstrated. However, the documentary distorted research results to attack the vaccine’s safety, resulting in decreased vaccination rates and lawsuits against vaccine manufacturers. These controversies resulted in a decline in pertussis vaccine uptake in the UK from 81% in 1974 to 31% in 1980, leading to an outbreak of pertussis in the UK, which placed significant strain and pressure on the National Health System [40]. The Academy of Pediatrics and the CDC continued to advocate for vaccination, hence, the overall controversy and public outcry had less impact on immunization rates in the US compared with in the UK. In response, the National Childhood Vaccine Injury Act of 1986 was passed so that vaccine-related injury claims could be filed while protecting vaccine manufacturers [25,40]. The National Vaccine Injury Compensation Program (NVICP) was also established by the US Department of Health and Human Services (DHHS) in 1988, requiring individuals alleging harm from a covered vaccine to first file a claim with the US Court of Federal Claims [41].

### 3.4. The Measles, Mumps, and Rubella (MMR) Vaccine Controversy

In 1998, Andrew Wakefield, a British gastroenterologist, and his colleagues published a report in *The Lancet,* alleging that the MMR vaccine caused autism in 12 children [42]. Despite the small case numbers and lack of supportive laboratory evidence, it sparked widespread media coverage, leading to a drop in MMR vaccination [43]. Large epidemiological studies over the following decade consistently found no evidence of a link between the MMR vaccine and autism [44,45,46]. Wakefield’s findings have never been replicated and, in 2004, he refused to join 10 of his co-authors in retracting the paper [47].

In 2010, *The Lancet* retracted the paper citing several inconsistencies. The UK’s General Medical Council (GMC) found that Wakefield’s subjects were carefully chosen, and some of Wakefield’s research was funded by lawyers for parents involved in lawsuits against vaccine manufacturers, revealing serious conflicts of interest [48].

In 1998, Wakefield highlighted that the MMR vaccine might be directly linked to inflammatory bowel disease and the subsequent development of autism in young children, and he believed that giving children only one dose of measles, mumps, and rubella may be safer [49]. Even as Wakefield sowed such fear, he was well aware that his own laboratory had refuted his central hypothesis that the measles virus caused bowel disease and autism [50]. Wakefield also filed patent applications with the London Patent Office nine months earlier for a new single measles vaccine, as well as various treatments and even “cures” for inflammatory bowel disease and autism. Scientists unanimously concluded that his ideas and suggested remedies lacked scientific credibility [49]. In 2006, *The Sunday Times* reported that Wakefield had been paid approximately half a million GBP by British litigation lawyers trying to establish that vaccination was harmful, with the hidden payments beginning two years prior to *The Lancet* paper [51].

In 2008, measles was declared endemic in England and Wales for the first time in 14 years. The UK’s Health Protection Agency linked this outbreak to a drop in MMR vaccination rates [52]. Wakefield’s personal campaign continued, going beyond initial MMR vaccines to criticizing the CDC in his controversial film *Vaxxed* [53]. Wakefield’s anti-vaccine stance is believed to have contributed to the 2015 California measles outbreak, infecting more than 130 individuals, as well as the 2017 outbreak in Minnesota [54].

### 3.5. Thiomersal Controversy

Thiomersal, used as a preservative in some vaccines, has also been at the center of the vaccination–autism controversy since the 1990s due to its mercury content [55]. The first serious concerns about its safety arose in the 1970s, as individuals became more aware of the dangers of organic mercury poisoning. The toxicity of ethylmercury used in thiomersal compared with methylmercury (the chemical linked to poisonings), which varies by one methyl side chain, is significantly lower. While the more toxic methylmercury is an industrial pollutant and can be found in fish, ethylmercury has been condemned by association despite the consensus view that it is safe when used in low concentrations [55].

Thiomersal is used in concentrations from 0.003% to 0.01% as a vaccine preservative [56]. In infants, the administration of vaccines containing thiomersal does not elevate mercury blood levels above safe levels [57]. Although there was no evidence that low thiomersal doses in vaccines caused any harm or for their supposed link with autism, in the US in 1999, the Public Health Service, the American Academy of Pediatrics, and vaccine manufacturers agreed that, as a precaution, thiomersal should be reduced or eliminated from vaccines. They also urged physicians to postpone the birth dose of hepatitis B vaccines in children not at risk of contracting the disease [58]. The decision to remove thiomersal sent mixed messages about vaccine safety; it also confused medical professionals and frightened parents, who then assumed that thiomersal was being targeted because it was harmful, breaking their trust in vaccine regulation. In 2000, parents formed several advocacy groups, believing that thiomersal was to blame for their children’s autism. Politicians also used the thiomersal controversy for political gain; in 2004, California governor, Arnold Schwarzenegger, prohibited thiomersal-containing influenza vaccines; other states, including Delaware, Illinois, Missouri, New York, and Washington, soon followed [59,60]. In the US, thiomersal was removed from all childhood vaccines by 2001, with the exception of the flu vaccine, which requires preservation as it is available in multi-dose vials [55].

**Figure 2:**
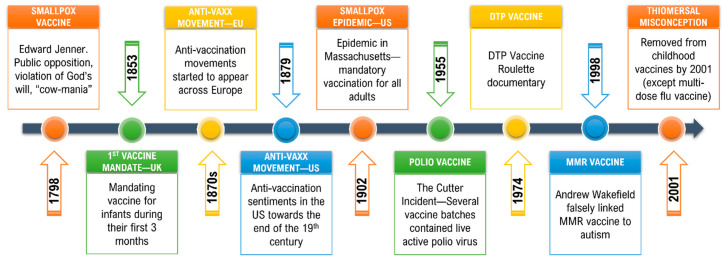
Vaccine hesitancy timeline: early hesitancy and modern controversies. Vaccination resistance existed since the discovery of vaccines, and public trust in their efficacy and overall safety has been challenged by various controversies up until the modern era.

The timeline of vaccine skepticism, from the early discoveries of vaccines to the controversies that surround vaccination in the modern era was depicted in Figure 2. 

## 4. Drivers of Vaccine Hesitancy

Decision making about vaccines and vaccination programs at a personal or populational level is complex, with a wide range of socio-demographic, attitudinal, and knowledge-based factors being important [61,62]. Attitudinal factors, such as one’s cultural and religious beliefs, the perception of risk or harm, and the behaviors of oneself and others, play critical and interconnected roles in decision making. Prior vaccine behavior and the impacts of vaccine mandates are additional factors that can predict vaccine uptake. COVID-19 vaccination has been specifically connected to political affiliation, ideological and partisan factors, information (including mis-information and dis-information), and satisfaction with government decision making on other aspects of COVID-19 prevention strategies and/or management [63].

### 4.1. Heuristics and Vaccine Hesitancy

The role of cognitive biases in human decision making is a key element that needs consideration. Individuals use heuristics to simplify specific problems when determining decisions [64]. A heuristic is a mental shortcut that allows people to solve issues and determine intuitive decisions rapidly [65]. If the correct variables initiate these heuristics, they can be quite useful. When a heuristic approach is employed while under the influence of wrong circumstances, it leads to systemic errors or cognitive biases in decision making [64].

The number of cognitive biases that affect vaccine hesitancy can be categorized into three groups based on the criteria that are common to each group [64]. Group one includes cognitive biases caused by vaccine-related information processing, which are strongly dependent on the message, its content, and pertinent aspects, such as the framing effect, availability bias, and authority bias. Group two involves cognitive biases triggered during vaccination decision making; uncertainty in the vaccine decision-making process, omission bias, risk perception of the decision maker, and ambiguity where there is preference to choose a known risk to an unknown one regardless of the outcome are among several factors that have the greatest influence on individual decisions. Group three cognitive biases have one thing in common: they all have a pre-existing belief about vaccination that has a bigger impact on actions than the content of the information that is available or provided. These examples include shared information bias, belief bias, and the false consensus effect [64].

### 4.2. Concerns Regarding Vaccine Safety and Efficacy

The success of vaccination programs means that fewer lay individuals and health professionals have first-hand experience or knowledge of the risks of vaccine-preventable diseases (VPDs). Consequently, attention is now often focused on the risk or perceived risk of vaccines or vaccination, rather than on the risk of infectious diseases [66].

The lack of trust in vaccination has been reinforced by several high-profile examples, in particular the paper by Wakefield, which proposed a link between autism and the MMR vaccine [67]. Although the paper was retracted and Wakefield’s findings proven to be flawed, it had long-term impacts on vaccine compliance [67].

Omission bias, the tendency to regard the negative consequences of an action (commission) as being worse than the negative consequences of inaction (omission) even when the latter affects more individuals or occurs more often [68], is suggested to play a role in vaccine hesitancy [69]. Some individuals believe it is more detrimental to vaccinate a child when the vaccination may cause harm than not to vaccinate, despite the fact that vaccination generally reduces the risk of harm [69]. One possible explanation for this decision is that vaccines are administered as preventive measures to healthy individuals; thus, their benefits can only be estimated accurately at the population level, whereas their risks (real or alleged) can be seen at the individual level [66].

### 4.3. Internet and Social Media Influence

The internet and social media are used to communicate, study, and influence individual and populational decisions regarding vaccines and vaccination. A study analyzed and identified the most common health mis-information issues on social media and found that 43% of mis-information was about vaccines [70]. Those who cite the internet as their primary source of vaccine information are more likely to refuse or delay vaccination [71,72,73]. Social media algorithms are designed to help users by filtering content to reflect an individual’s prior search patterns. It predicts and determines decisions on which material would be best to offer based on one’s prior behavior. As a result, if a person searches or follows sites or individuals that are linked to vaccine hesitancy or refusal, they are more likely to see similar content in their future searches [74]. Additionally, the internet does not determine much distinction between the quality and reliability of information provided by a healthcare expert or a lay person [74]. Hence, the internet and social media platforms have become major channels for the distribution of inaccurate data or mis-information about vaccines and vaccinations, and this is widely exploited [75,76,77,78]. For example, from surveys on social media platforms in the US and UK, it is known that there has been a widespread dissemination of false information about the pandemic, including that 5G mobile networks are linked to the virus, that vaccine trial participants have died after receiving a candidate COVID-19 vaccine, and that the pandemic is a conspiracy or a bio-weapon [79,80,81]. Such information can exacerbate pre-existing anxieties, sowing doubts and cynicisms about new vaccines and threatening the public acceptance of vaccines.

### 4.4. Mandatory Vaccination and Public Health Policies

Preserving vaccine-acquired herd immunity against an infectious disease requires vaccination coverage above particular threshold values in a population [82]. Vaccination policies vary considerably around the globe; some countries emphasize educating their citizens and allowing individuals to choose, while others mandate immunizations to ensure a high vaccine coverage. A good example for the latter is mandating vaccination in children for a number of diseases as a condition of school entry [83]. Nonetheless, despite being effective, mandatory vaccination policies have long been controversial, widely criticized as an authoritarian measure, and are not accepted by some individuals or in some societies [84].

According to Kennedy et al. [85], a parent’s objection to mandatory vaccination is linked with negative attitudes and views towards the safety and efficacy of vaccines. Additionally, a vaccine mandate induces frequently observed reflections, such as “it is my body, my choice”, as well as widely accepted notions such as “the child’s body can protect itself without vaccination”, referring to a natural immunity being better than the immunity from vaccination. This is only true for some diseases. Disease severity varies among individuals, but some infections, such as rabies, can be fatal in almost all cases. In the absence of lived experience, some parents underestimate the direct threat to their child’s health of contracting VPDs [85]. Another US study by McCoy regarding opposition to vaccine mandates suggested that social factors that form Americans’ opinions about vaccine safety are not the same as the factors that determine their views on whether vaccination should be compulsory. The reason may potentially be due to some conservatives who hold political beliefs that governments should play a limited role and the primacy of individual liberty/freedom [86].

### 4.5. Vaccine Hesitancy in the COVID-19 Era

Vaccine hesitancy has been the subject of much discussion as vaccines have become available against COVID-19. Factors predictive of openness to being vaccinated for COVID-19 (in rank order) include the perceived safety of vaccines, perceived risk of COVID-19, perceived effectiveness of vaccines, political ideology, trust in medical professionals, and trust in scientists. Conversely, factors predictive of the refusal to be vaccinated (in rank order) include the perceived safety of vaccines, political ideology, trust in medical professionals, ethnicity, perceived moral reproach, perceived effectiveness of vaccines, and social media reliance for information [87]. Hence, it seems that those open to receiving vaccines are more likely to base their decisions off of weighing up risks and benefits, and those who are vaccine-hesitant are more likely to be influenced by other attitudes and beliefs.

Frequently cited reasons for vaccine hesitancy include skepticism about the impartiality of some of the research, the rapid development of vaccines, and their short-term and unknown long-term side effects. The lack of trust in research is possibly due to a level of uncertainty and constantly evolving scientific findings. This situation is not new or unusual, but it was unusual for this early information to be so readily available to the public, often without interpretation by a scientific or medical intermediary. Furthermore, for decades, evidence-based medicine paradigms and approaches to therapeutics have recommended a slow and staged introduction and the adoption of new medications, highlighting the fact that randomized controlled trials are better at showing efficacy than elucidating adverse effects. In this pandemic, the community was asked to re-assess the risk–benefit equation, given the significant risk of infection and subsequent serious harm from the virus.

The link between the AstraZeneca vaccine and blood clots was initially denied by several regulatory agencies before being clearly shown to exist. This change of advice may have reduced confidence in the regulatory process. In addition, many regulators, in the interest of transparency, announced all early cases of vaccine-associated clotting, which may have disproportionately heightened the awareness of a rare side effect. Similarly, the incidence of myocarditis in children secondary to the Pfizer vaccine alarmed parents who were also advised that COVID-19 is usually a mild disease in children.

The politization of pandemic responses and other measures, inconsistencies between policies, and attitudes of key political leaders and decision makers also exacerbated the situation. Various conspiracy theories have emerged around the pandemic and vaccines, as well as the source of virus, reasons behind lockdowns around the globe, and mandatory vaccinations. In this context, media communication, particularly social media, seems to have played an important role. In one study, social media was linked to anxieties about the COVID-19 pandemic, as well as vaccine reluctance and related conspiracy theories [88]. The novelty of the disease, particularly the lack of knowledge about emerging variants of the virus; vaccines based on new platforms such as mRNA and vector-based vaccines; doubts about the efficacy of existing vaccines against new variants; political and economic motives attributed to the pandemic response, vaccine development, and manufacturing; and public distrust in medical companies and governments have all contributed to growing uncertainty over COVID-19 vaccination [3,89].

For example, in December 2021, Robert Malone, the self-proclaimed “inventor of mRNA vaccination,” contributed a series of claims about the mRNA COVID-19 shots harming children in a video viewed hundreds of thousands of times online. This video was later repudiated [90] and Twitter permanently suspended Malone’s account for violating the platform’s policies regarding COVID-19 mis-information [91].

Joe Rogan, an actor and comedian, interviewed Roger Malone on 31 December 2021 for The Joe Rogan Experience, a Spotify-exclusive podcast. Malone shared numerous inaccurate, misleading, and unsupported claims concerning the safety and immunity of COVID-19 treatments and vaccines [91]. Malone’s assertions during Rogan’s interview prompted 270 scientists, medical experts, educators, and science communicators to co-sign an open letter urging Spotify to “mitigate the propagation of disinformation on its platform.” [92].

## 5. Vaccine Hesitancy across Different Countries, Cultures, and Socio-Economic Groups

**Figure 3:**
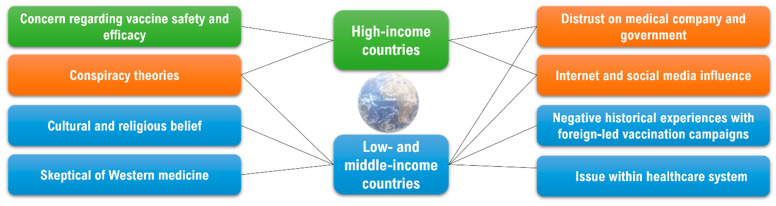
Drivers’ vaccine hesitancy in HICs and LMICs. Concern about vaccine safety and efficacy are most common in HICs (in green), meanwhile cultural and religious beliefs, negative historical experiences with foreign medicine and vaccination campaign, as well as issues within healthcare system are usually common in LMICs (in blue). Other factors such as distrust in medical companies and governments, conspiracy theories, and social media mis-information are common in both categories (in orange).

Vaccine hesitancy is linked to a variety of socio-economic and demographic factors. Concerns about vaccine safety and efficacy are most common in high-income countries (HICs), whereas in low- and middle-income countries (LMICs), cultural and religious beliefs, negative historical experiences with foreign medicine and vaccination campaigns, as well as issues within healthcare systems are more common. Other factors that are common in both categories include distrust in medical companies and the government, conspiracy theories, and social media mis-information (Figure 3).

### 5.1. Vaccine Hesitancy in High-Income Countries (HICs)

Concerns about vaccination safety are a major reason for the current vaccine hesitancy in HICs. In 2009, Denmark was one of the first countries to publicly fund HPV vaccination to 12-year-old girls, and the program was a success during the first year following its launch (>90 percent of girls born from 1998 to 2000 received at least one dose of the HPV vaccine) [93]. From 2013, the program faced increasing scrutiny from the media as some adverse events were reported. The Danish media began reporting alleged adverse events involving Danish girls and women, including a documentary in March 2015 describing a group of girls with a variety of disabling symptoms presumed to be caused by HPV vaccination. Although no epidemiological study has been able to prove a link between HPV vaccination and increased risk of adverse events, there was a rapid decline in HPV vaccination uptake from 90% for girls born in the period 1998 to 2000 (in 2009) to 54% for girls born in 2003 (in 2014) [93]. Similarly, in Ireland, the decline in HPV vaccination was caused by parental concerns about vaccine safety, which were spread by advocacy groups formed in 2015. With the help of local and national media, these groups established a strong social media platform with emotive personal narratives, lobbied politicians, and disseminated mis-information. After media reported on unconfirmed adverse reactions to the HPV vaccine, the Japanese Ministry of Health, Labor, and Welfare suspended pro-active recommendations in June 2013. In January 2014, the Vaccine Adverse Reactions Review Committee concluded that there was no evidence for a causal relationship between the HPV vaccine and reported adverse events; however, they did not reinstate the pro-active recommendations for its use [94].

France was one of the world’s most vaccine-hesitant countries, even before the COVID-19 pandemic. Approximately half (41%) of French citizens have doubts about vaccine safety, compared to a global average of 13% [95]. Despite the high skepticism, the anti-vaccination movement in France has never been particularly strong until the first major vaccine concern appeared towards the end of the 1990s following a significant vaccination program against hepatitis B when community members and nurses expressed concerns about a possible link between hepatitis B vaccination and multiple sclerosis. Vaccination rates against this disease rapidly declined, and the initiative was halted in 1998 after only four years [96]. In 1999, the WHO established the Global Advisory Committee on Vaccine Safety (GACVS) to promptly respond and provide the unbiased scientific assessment of vaccine safety issues; later, the above-mentioned claims were debunked [97]. By 2004, the vaccine’s alleged risks had faded from the headlines, and vaccination rates increased since then, due in part to the inclusion of the hepatitis B strain in the multivalent pediatric DT-IPV and other vaccines [96]. Media in France had critical influence on the rise of vaccination reluctance. Its rapid surge coincided with the public controversy in 2009 over the influenza pandemic A/H1N1 vaccination campaign. The French government sought to vaccinate 70% of the general population and spent almost a billion EUR to combat the virus [96]. Even before vaccinations began, heated disputes in the media erupted over a range of aspects of this policy, including its expense and the vaccine’s safety. In the end, only approximately 8% of French citizens received vaccinations. Since then, the subject of vaccination safety has almost constantly been in the headlines, with the media voicing concerns about the HPV vaccine, multi-valent vaccines, and the use of aluminum-based adjuvants [98].

When COVID-19 vaccines were introduced in France, President Emmanuel Macron intervened in response to these concerns of vaccine hesitancy. He urged his citizens to get vaccinated and announced a series of measures, including mandatory COVID-19 vaccinations for healthcare personnel, and the requirement to show a *passe sanitaire* (health pass) to enter public events. Despite some criticisms, the French parliament passed the legislation supporting these measures and they were widely implemented during the summer, and France quickly began to improve compared to average vaccination rates in the EU. Within two months, 91.4% of France’s population had received their first dose, compared to 76.6% in the EU [99].

With COVID-19 vaccination campaigns underway in many countries, surveys have indicated significantly higher vaccine hesitancy among some ethnic minorities [100,101,102]. For example, vaccine hesitancy was highest among Black/Black British, Bangladeshi/Pakistani populations in a UK survey in December 2020, compared to people of Caucasian ethnic origin [103]. Concerns about safety and long-term health effects, as well as a lack of trust in vaccines and the pharmaceutical industry, were the most common reasons for hesitancy, particularly among Black/Black British respondents [103]. Some used these fears to spread mis-information, adding to the historical mis-trust of the government and public health agencies that exist among some groups [104]. This mis-trust was exacerbated by systemic racism and discrimination [105], such as previous unethical health research in Black/Black British populations [106], the under-representation of minority groups in research and vaccine trials, and unpleasant experiences in culturally insensitive healthcare systems and services [105,107].

Six months before the national COVID-19 vaccine rollout, over 3000 Australian adults were asked in an online survey whether they would accept COVID-19 vaccination if a safe and effective vaccine was available. The responses were 5.5% “definitely not”, 7.2% “probably not”, 28.7% “probably will”, and 58.5% “will” [108]. A follow-up with the same individuals in January, immediately prior to the vaccine rollout in February 2021, revealed a slight trend towards vaccine hesitancy, with 7.8% choosing “definitely not” and 13.2% choosing “probably not”. Although vaccine willingness remained relatively high, 82% of respondents expressed concerns about possible side effects, particularly associated with the AstraZeneca vaccine [109]. One commentator noted that hesitancy increased in April and May because of the publicity surrounding blood clots, but subsequently decreased as the vaccination program progressed. It decreased even more sharply in July 2021, when larger outbreaks in NSW and Victoria began [110]. According to the Melbourne Institute’s Vaccine Hesitancy Tracker, vaccine hesitancy in Australia continued to fall, from 15% in September to 13.3% in October 2021, and then to 9.6% in December 2021 [111].

### 5.2. Vaccine Hesitancy in Low- and Middle-Income Countries (LMICs)

Vaccine hesitancy is also a major problem in LMICs. In addition to concerns about the harmful effects of vaccines/vaccination, the matter is influenced by social, cultural, and religious beliefs.

The fear of vaccine-associated harm was the most frequently reported concern in a 2014 mixed-methods study by Qutaiba et al. [112]. A Kyrgyzstan study found that while 3% of respondents thought vaccinations were unsafe, 62% thought they weakened the immune system [113]. In Nigeria, 33% of respondents stated that the polio vaccination could harm the child [114,115]. Parents who had not vaccinated their children or had a negative attitude toward vaccination were more likely to express such anxieties [116]. The first reason given by parents in India, Nigeria, and Pakistan for not vaccinating their children was also the potential harmful nature of vaccines [116,117,118,119]. The fear of serious adverse effects could also be a result of previous experiences with adverse events following immunization (AEFI), which could be attributed to the vaccination event [120], that vaccinations led to adverse reactions such as fever added to the assumption that vaccines could be harmful [121]. In Pakistan, one of the most frequently reported speculations was that the polio vaccine caused adult sterility, resulting in many parents refusing to vaccinate their children [122].

Concerns about vaccines and immunization programs also stem from the belief that vaccines are part of a global conspiracy against certain communities such as Africans, dark-skinned people, some minorities, or religious faiths, including Islam and Christianity [116]. For example, in 2003–2004, a boycott of polio vaccination occurred in five Muslim-majority states of northern Nigeria—Kano, Zamfara, Kaduna, Niger, and Bauchi—after religious and political leaders endorsed rumors that the oral polio vaccine was part of an American plot to spread HIV and cause infertility. The rumors had already been circulating for many years and the political tensions in the April 2003 elections provided an opportunity for northern state governments to complicate matters for the federal government [123]. Although the veracity of the rumors was never proven, the general public in northern Nigeria remained skeptical of Western medicine [124]. Pakistan nearly eradicated polio by the mid-2000s. In 2005, 28 cases (1.4% of global total) were reported. Local political officials did not intervene with polio workers throughout this time. However, the drone strikes operation and a fake hepatitis B vaccination campaign by the CIA in 2011 to collect DNA from Osama bin Laden’s relatives to learn more about his whereabouts prior to Operation Neptune Spear [125] had long-term consequences in Pakistan’s northwestern corner [126], raising concerns about the motives behind vaccination campaigns, including polio, resulting in an increase in polio cases from 198 in 2011 to 306 in 2014, accounting for 85% of all cases of polio worldwide [127,128]. At least 70 polio workers have been killed in Pakistan since 2012, with the Taliban claiming responsibility for many of the attacks, stating that the vaccination campaigns were a cover for spy or intelligence gatherings [129]. Public health and medical aid organizations all over the world criticized the CIA’s campaign for the negative impact it has had on Pakistan’s public health initiatives [126].

A study in Christian areas in Parakou and Cotonou, West Africa, identified that some parents’ refusal to vaccinate their children was motivated by their beliefs in the miracle of prayer. Their religious leaders claimed that vaccines caused healthy children to become sick. Vaccination, they believed, was against God’s will, thus, equivalent to making a *“deal with the Devil”,* and was viewed as *“the work of the white witch doctor, in violation of Biblical scriptures”.* The faithful Cotonou’s churches stated, unequivocally, that vaccines were poisons created by white people to harm and conduct experiments on people of color by infecting them with diseases [130]. Most Nigerian communities are riddled with myths and misconceptions about vaccination due to poor knowledge and superstitions about VPDs [131].

Issues within healthcare systems also contribute to vaccine hesitancy in LMICs. Insensitive healthcare workers [121], the fear of being ‘scolded’ by the vaccinator for losing the immunization card and having to pay for a new one [131], as well as other costs related to receiving immunization and the scarcity of vaccination centers resulting in individuals having to travel long distances to get vaccinated were among major impediments to receiving vaccines [132].

A recent study on vaccine acceptance revealed that people in LMICs in Asia, Africa, and South America appeared more willing to receive the COVID-19 vaccine than those in Russia and the US. Nonetheless, vaccine hesitancy persists, with the most common reason being that vaccines are unsafe, as seen in Burkina Faso. The relatively low acceptance rate in Pakistan, on the other hand, may be attributed to negative historical experiences with foreign-led vaccination campaigns [133].

## 6. Measures to Counteract Vaccine Hesitancy

### 6.1. Public Education and Communication—Multi-Cultural and Multi-Lingual

The COVID-19 pandemic has resulted in enormous amounts of information for the public, and a parallel spread of inaccurate and misleading information has intensified public confusion and anxiety. COVID-19 mis-information and dis-information must be dealt with swiftly and effectively through simple, consistent, persistent, and strong counter-messaging measures, which are necessary to build confidence in vaccines and vaccination programs. Collaboration across many socio-economic sectors, not only in healthcare, and with communities that are more likely to be affected is important to such unbiased and objective information dissemination efforts. Such communications about vaccine safety and population health benefits of vaccinations must be delivered by individuals who are trusted by the community, for example, high-profile role models, as well as individuals who are able to answer questions and queries in an objective and non-judgmental manner, such as doctors, nurses, pharmacists, or scientists working in a relevant area of healthcare or science [134].

Vaccine manufacturers, as well as governments and health organizations, both nationally and internationally, must continue to be advocate for the spread and promotion of positive public health beliefs and trust necessary for mass vaccination acceptance globally. This includes the sharing of scientific information openly and transparently, as well as the promotion of benefits that COVID-19 vaccination offers and any potential adverse side effects, their severity, and likely frequency [10,134].

Watson et al. utilized mathematical modelling to quantify the direct and indirect global impact of COVID-19 vaccination. Using their model fit to predict and report on excess mortality, the authors estimated that 31.4 million deaths due to COVID-19 would have occurred without vaccinations during the first year of COVID-19 vaccination, with 19.8 million deaths averted, corresponding to 63% (19.8 million of 31.4 million) of total deaths. The study found that 96 countries and administrative regions were below the WHO target of 40% vaccination coverage by the end of 2021. Had this target been met, they estimated that 599,300 additional deaths would have been averted. The majority of these deaths occurred in low- and middle-income countries and the African and Eastern Mediterranean regions, although the largest proportional increase was seen in LMICs, with the averted deaths making up a 111% increase in estimated deaths averted through vaccination [135].

### 6.2. Behavioral Interventions and Catalysts for Change

For over two decades, behavioral scientists have been examining strategies to increase vaccine acceptance among parents of children and adolescents, particularly for measles, rubella, and mumps or human papillomavirus vaccinations, and among adults particularly for influenza, pneumonia, and shingles vaccinations [136,137,138]. This has generated a substantial body of knowledge on vaccine decision-making processes, which identified successful public health pathways for COVID-19 vaccination advocacy during this pandemic [136,139,140].

Promoting behavior change to encourage vaccination acceptance and uptake is a complex process that incorporates various social impacts on individual decision making and requires coordinated activity at multiple levels by individuals. They range from policymakers who determine decisions for their jurisdictions (states/provinces/regions/countries), organizational leaders (religious leaders, healthcare professionals, company executives) who may be influential among their followers/patients/employees, and lay citizens who, by accepting (or refusing) vaccination, ultimately contribute to the success (or failure) of global vaccination efforts. More importantly, both the source of the message and the emotions it elicits determine the effectiveness of these interventions [136].

Social and behavioral sciences can provide useful insights into how to manage the pandemic and its implications. It can assist policymakers, leaders, and the public in providing a greater understanding of how to manage threats, explore various social and cultural aspects, achieve better scientific communication, implement effective leadership, and provide social and emotional support [141].

A range of behavioral interventions known as nudge interventions have been shown to be effective for improving vaccine uptake, including text messages for influenza vaccination in pharmacies [142] and mailed reminder letters [143].

### 6.3. Importance of Political Will and Leadership

Politicians and public figures are critical in engendering and sustaining public trust in public health measures, especially during a pandemic. How governments respond to disease outbreaks in general has a significant impact on public trust in regard to vaccination. The influence of politicians on public opinion is arguably larger than that of celebrities, as they can actively impact health policies and influence attitudes. They bear an obligation to offer accurate and unbiased health information, and not doing so can lead to increased concerns about vaccination.

For example, the MMR vaccine–autism controversy that arose in 2001 in response to the debunked Wakefield study was exacerbated when the then UK Prime Minister, Tony Blair, refused to disclose his own child’s vaccination status, which caused parents to be afraid and anxious, and slowed down public health efforts [144]. President Trump’s early 2017 call for a vaccine safety commission chaired by vaccine sceptics Robert Kennedy Jr and Robert De Niro concerned health professionals worldwide [145]. In March 2017, Senator Pauline Hanson sparked outrage in Australia by promoting a non-existent ‘vaccination reaction test’, a claim which she later withdrew [146].

The Vaccine Confidence Project conducted research across 32 countries, and in December 2020 reported that confidence in governments’ handling of the COVID-19 reaction was the strongest indicator of willingness to accept a COVID-19 vaccine [147]. Those who thought their government was handling the pandemic response well were more willing to receive COVID-19 vaccination; those who thought their government was handling it poorly were less willing [148].

### 6.4. Pivotal Input from Healthcare Providers/Clinicians

A committed, confident, and knowledgeable vaccination workforce is critical to achieving a high vaccine coverage. Physicians’ and public health professionals’ confidence can be helpful in diminishing or overcoming anxieties about vaccines and vaccinations. If health professionals have a comprehensive understanding of vaccinations and vaccine hesitation, they are more equipped to guide parents and patients in their vaccine decision making [149]. Clinician recommendations appear to be one of the most effective methods for increasing vaccination rates. Since healthcare workers are the most knowledgeable in the relevant field and are among the first to receive vaccinations, they should be able to speak authoritatively and confidently to their patients about their decision to receive vaccines themselves and encourage their patients and families to also receive the vaccines [150].

Clinicians can implement a variety of approaches to overcome vaccine hesitancy. These include knowing the vaccination schedule, providing clear recommendations, adopting standing orders, or nursing protocols to deliver vaccinations without clinical examinations, as well as reaching out to patients through reminders and recalls between visits. The Corroborate, About Me, Science, and Explain/Advised (C.A.S.E.) approach is one of many promising ways to address vaccine hesitancy, where the clinician begins by identifying the nature of the hesitancy and then establishing a common aim or aspiration and providing reassurance in a non-judgmental way to the hesitant patient. Clinicians must demonstrate command of both experience and evidence. Following that, the clinician should outline the relevant information and explain/advise in terms of the common ground and the underpinning science in lay terms, ensuring that the patient understands the suggestion in a way that addresses the patient’s hesitation in the context of the patient’s belief systems [11].

### 6.5. Mandatory Vaccinations

There is substantial literature for mandatory vaccination, particularly to mitigate infectious diseases. One of the most common arguments put forward by bio-ethicists focusses on preventing harm to others [151,152]. In 1859, the philosopher John Stuart Mill argued that individuals should be free in making decisions if their actions are not harmful to others, also known as the *harm principle.* If their decisions harm others, then governmental coercion and the restriction of liberty are justifiable [153]. Mandatory vaccination can be ethically justified if the following conditions are met: the threat to public health is grave, the confidence in safety and effectiveness is high, the predicted value/benefit exceeds the alternatives, and the coercion is applied in a proportional manner (e.g., non-compliance penalties or costs are reasonable) [154].

Compulsory childhood vaccination has become a significant policy intervention for governments seeking to overcome low immunization rates. Childhood immunizations include those that protect against measles, mumps, rubella, diphtheria, tetanus, pertussis, polio, hepatitis B, rotavirus, Hemophilus influenzae type B, and tuberculosis—several of these are combination vaccines. Mandatory vaccinations are required for a specific purpose, most commonly school entry for children; thus, it is important to gain a thorough understanding of policies in different countries [155].

Some countries have mandated or are considering mandating COVID-19 vaccine certification, which requires proof of at least two doses of an approved vaccine, a negative viral test, or a recovery certificate to demonstrate a recent natural infection. As already mentioned (Section 5), one such initiative is the French government requirement for a health pas*s* or *passe sanitaire* [156]. Other countries which have mandated or plan to mandate COVID-19 vaccination for all adults (except individuals with medical exemptions) include Ecuador, Germany, Indonesia, and Micronesia. Canada, Costa Rica, Denmark, Egypt, Italy, New Zealand, Saudi Arabia, and many other countries require vaccination for health workers, government employees, and other public and private sector workers [157].

## 7. Conclusions and Future Outlook

Vaccine hesitancy and anti-vaccine movements have existed since vaccines were first discovered and became commercially available. The rise in controversies surrounding vaccines and vaccination programs, as well as outbreaks of vaccine-preventable diseases in unvaccinated or under-vaccinated populations, has heightened public health concerns. Although research data have been available in the last decade identifying the causes, consequences, and impacts of vaccination hesitancy, there is still an urgent need to further understand and address public apprehension about vaccines. As we slowly emerge from the last two years of COVID-19 and health restrictions, it is even more critical to conduct focused research on vaccine hesitancy to obtain a deeper and more thorough understanding of community dynamics, socio-cultural influences, and indigenous knowledge and beliefs, as well as how criticisms about vaccines and vaccinations; whether valid, false, or perceived to be true; can affect vaccination acceptability, and how to overcome these concerns in a non-judgmental and measured manner.

## Data Availability

Not applicable.
